# Supplementary material for: Immunological imprinting shapes the cross-reactive antibody responses to the KP.2 and LP.8.1 vaccine doses
Source: J Virol. 2026 Aug 26;100(9):e00749-26. doi: 10.1128/jvi.00749-26 (PMC13595941; doi:10.1128/jvi.00749-26)
Supplement: Supplementary file 1 [file jvi.00749-26-s0001.docx]

**SUPPLEMENTARY MATERIALS**

**LIST OF SUPPLEMENTARY MATERIALS**

Fig. S1

Table S1 to S2

**Figure S1.**

**Fig. S1. Nucleocapsid-specific antibody responses during the six-month follow-up after KP.2 mRNA vaccination.** IgG antibody binding to the SARS-CoV-2 nucleocapsid (N) protein measured by electrochemiluminescent immunoassay across the longitudinal sampling period. Antibody levels are shown for a subset of participants (n = 10) included for durability analysis at baseline prior to KP.2 vaccination (pre-vaccination) and at 1 month (1M), 3 months (3M), and 6 months (6M) after vaccination. Each symbol represents an individual participant and lines connect longitudinal samples from the same individual. Antibody concentrations are reported as arbitrary units per milliliter (AU/mL) based on normalization to a standard reference curve.

**Supplementary Table S1: Demographic information for individuals in the study group**

**Supplementary Table S2: Amino acid substitutions in spike protein of variants used in the study**

| **Variant** | **Amino acid substitutions** | **Virus Name** | **GISAID** |
| --- | --- | --- | --- |
| A |  | SARS-CoV-2/human/USA/WA-CDC-WA1-F6/2020 | MT020881 |
| BA.5.5 | I19T, S24L, -25P, -26P, -27A, -69H, -70V, I76T, D142G, G213V, D339G, F371S, P373S, F375S, A376T, N405D, S408R, N417K, K440N, R452L, N477S, K478T, A484E, V486F, R498Q, Y501N, H505Y, G614D, Y655H, K679N, H681P, K764N, Y796D, H954Q, K969N | hCoV-19/USA/COR-22-063113/2022 | EPI_ISL_13512579 |
| XBB.1 | I19T, S24L, -25P, -26P, -27A, A83V, D142G, -144Y, Q146H, E183Q, E213V, V252G, H339G, T346R, I368L, F371S, P373S, F375S, A376T, N405D, S408R, N417K, K440N, P445V, S446G, K460N, N477S, K478T, A484E, P486F, S490F, R498Q, Y501N, H505Y, G614D, Y655H, K679N, H681P, K764N, Y796D, H954Q, K969N | hCoV-19/USA/MD-HP40900-PIDYSWHNUB/2022 | EPI_ISL_16026423 |
| KP.2 | I19T, T21R, S24L, -25P, -26P, -27A, L50S, -69H, -70V, F127V, D142G, -144Y, S157F, G158R, R187K, -211N, I212L, G213V, F216L, N245H, V332I, H339G, T346R, T356K, F371S, P373S, F375S, A376T, K403R, N405D, S408R, N417K, K440N, H445V, S446G, D450N, W452L, S455L, L456F, K460N, N477S, K478T, K481N, -483V, K484E, P486F, R498Q, Y501N, H505Y, K554E, V570A, G614D, S621P, Y655H, K679N, R681P, K764N, Y796D, F939S, H954Q, K969N, L1104V, L1143P | hCoV-19/USA/CA-Stanford-181_S33/2024 | EPI_ISL_18993067 |
| BA.2.86 | I19T, T21R, S24L, -25P, -26P, -27A, V35G, L50S, -69H, -70V, F127V, D142G, -144Y, S157F, G158R, -211N, I212L, G213V, F216L, N245H, D264A, V332I, H339G, T356K, F371S, P373S, F375S, A376T, K403R, N405D, S408R, N417K, K440N, H445V, S446G, D450N, W452L, K460N, N477S, K478T, K481N, -483V, K484E, P486F, R498Q, Y501N, H505Y, K554E, V570A, G614D, S621P, Y655H, V670I, K679N, R681P, K764N, Y796D, F939S, H954Q, K969N, L1143P | SARS-CoV-2/human/USA/MI-UM-10052670540/2023 | OR537351 |
| KP.3.1.1 | I19T, T21R, S24L, -25P, -26P, -27A, -31S, L50S, -69H, -70V, F127V, D142G, -144Y, S157F, G158R, -211N, I212L, G213V, F216L, N245H, D264A, V332I, H339G, T356K, F371S, P373S, F375S, A376T, K403R, N405D, S408R, N417K, K440N, H445V, S446G, D450N, W452L, S455L, L456F, K460N, N477S, K478T, K481N, -483V, K484E, P486F, E493Q, R498Q, Y501N, H505Y, K554E, V570A, G614D, S621P, Y655H, K679N, R681P, K764N, F939S, H954Q, K969N, L1104V, L1143P | hCoV-19/USA/NY-GBW-GKISBBBC17729/2024 | EPI_ISL_19191532 |
| XEC | I19T, T21R, N22T, S24L, -25P, -26P, -27A, L50S, S59F, -69H, -70V, F127V, D142G, -144Y, S157F, G158R, -211N, I212L, G213V, F216L, N245H, V332I, H339G, T356K, F371S, P373S, F375S, A376T, K403R, N405D, S408R, N417K, K440N, H445V, S446G, D450N, W452L, S455L, L456F, K460N, N477S, K478T, K481N, -483V, K484E, P486F, E493Q, R498Q, Y501N, H505Y, K554E, V570A, G614D, S621P, Y655H, K679N, R681P, K764N, Y796D, F939S, H954Q, K969N, L1104V, L1143P | hCoV-19/USA/CA-Stanford-195_S33/2024 | EPI_ISL_19456412 |
| LP.8.1 | I19T, T21R, S24L, -25P, -26P, -27A, -31S, L50S, -69H, -70V, F127V, D142G, -144Y, S157F, G158R, L186F, S190R, -211N, I212L, G213V, F216L, N245H, D264A, V332I, H339G, T346R, T356K, F371S, P373S, F375S, A376T, K403R, N405D, S408R, N417K, K440N, R445V, S446G, D450N, W452L, S455L, L456F, K460N, N477S, K478T, K481N, -483V, K484E, P486F, E493Q, R498Q, Y501N, H505Y, K554E, V570A, G614D, S621P, Y655H, K679N, R681P, K764N, Y796D, F939S, H954Q, K969N, R1086K, L1104V, L1143P | hCoV-19/USA/CA-GBW-GKISBBBE30320/2024 | EPI_ISL_19531918 |
| LF.7 | I19T, T21R, N22T, S24L, -25P, -26P, -27A, P31S, L50S, -69H, -70V, F127V, D142G, -144Y, S157F, G158R, R182K, S190R, -211N, I212L, G213V, F216L, N245H, D264A, V332I, H339G, T346R, T356K, F371S, P373S, F375S, A376T, K403R, N405D, S408R, N417K, K440N, R444K, H445V, S446G, D450N, W452L, S455L, L456F, K460N, V475A, N477S, K478T, K481N, -483V, K484E, P486F, R498Q, Y501N, H505Y, K554E, V570A, G614D, S621P, Y655H, K679N, X681P, K764N, Y796D, F939S, H954Q, K969N, L1143P | hCoV-19/USA/VA-GBW-GKISBBBD55343/2024 | EPI_ISL_19451838 |
| XFG.3.12 | I19T, T21R, N22T, S24L, -25P, -26P, -27A, P31S, L50S, -69H, -70V, F127V, D142G, -144Y, S157F, G158R, R182K, S190R, -211N, I212L, G213V, F216L, N245H, D264A, V332I, H339G, T346R, T356K, F371S, P373S, F375S, A376T, K403R, N405D, S408R, N417K, K440N, R444K, R445V, S446G, D450N, W452L, S455L, L456F, K460N, N477S, K478T, K481N, -483V, K484E, P486F, D487N, E493Q, R498Q, Y501N, H505Y, K554E, V570A, I572T, G614D, S621P, Y655H, K679N, R681P, K764N, Y796D, F939S, H954Q, K969N, L1143P | hCoV-19/USA/NY-GBW-GKISBBBF07468/2025 | EPI_ISL_19824940 |
| PQ.1  (NB.1.8.1) | I19T, T21R, N22T, S24L, -25P, -26P, -27A, L50S, S59F, -69H, -70V, F127V, D142G, -144Y, S157F, G158R, S184G, -211N, I212L, G213V, F216L, N245H, D264A, V332I, H339G, T356K, F371S, P373S, F375S, A376T, K403R, N405D, S408R, N417K, S435A, K440N, H445V, S446G, D450N, W452L, S455L, L456F, K460N, N477S, I478T, K481N, -483V, K484E, P486F, E493Q, R498Q, Y501N, H505Y, K554E, V570A, G614D, S621P, Y655H, K679N, R681P, K764N, Y796D, F939S, H954Q, K969N, L1143P | SARS-CoV-2/human/USA/MA-CDC-5923231-001/2025 | PV812494 |
| BA.3.2.1 (RD.1.1) | L9P, T21R, L26P, V67A, -69H, -70V, I95T, T101I, -136C, -137N, -138D, -139P, -140F, -141L, -142G, -143V, -144Y, -145Y, -146H, -147K, S157F, K164N, F172S, T187K, A211N, S212L, D213V, T214R, L227V, -243A, -244L, S251P, V326I, Y339G, P348A, F371S, P373S, F375S, K403R, N405D, S408R, N417K, S435A, R440N, A445V, D446G, W452L, K460N, N477S, N478T, K484E, S496G, R498Q, Y501N, N529K, D554E, D583E, G614D, R625H, K641N, G642V, K654E, Y655H, R679N, R681P, D688A, L704S, K764N, T795K, Y796D, K852A, F939S, H954Q, K969N, R1162P, E1184D | hCoV-19/USA/CA-GBW-GKISBBBG92145/2025 | EPI_ISL_20054259 |
| RE.2  (BA.3.2.2) | L9P, T21R, L26P, V67A, -69H, -70V, I95T, T101I, -136C, -137N, -138D, -139P, -140F, -141L, -142G, -143V, -144Y, -145Y, -146H, -147K, S157F, K164N, F172S, T187K, -211N, I212L, -243A, -244L, S251P, V326I, Y339G, P348A, T356K, F371S, P373S, F375S, K403R, N405D, S408R, N417K, S435A, R440N, A445V, D446G, W452L, K460N, N477S, N478T, K484E, S496G, R498Q, Y501N, N529K, D554E, S575A, D583E, G614D, R625H, K641N, G642V, K654E, Y655H, R679N, H681P, D688A, L704S, K764N, T795K, Y796D, K852A, F939S, H954Q, K969N, E1184D | hCoV-19/USA/NY-GBW-GKISBBBI08250/2025 | EPI_ISL_20262473 |
